# Tripartite efflux pumps: energy is required for dissociation, but not assembly or opening of the outer membrane channel of the pump

**DOI:** 10.1111/mmi.12211

**Published:** 2013-04-09

**Authors:** Thamarai K Janganan, Vassiliy N Bavro, Li Zhang, Maria Inês Borges-Walmsley, Adrian R Walmsley

**Affiliations:** 1 School of Biological and Biomedical Sciences, Durham UniversitySouth Road, Durham, DH1 3LE, UK; 2 Institute of Microbiology and Infection, School of Immunity and Infection, University of BirminghamEdgbaston, Birmingham, B15 2TT, UK; 3 Department of Molecular Biology and Biotechnology, University of SheffieldUK; 4 Department of Molecular Biology, Swedish University of Agricultural SciencesSweden

## Abstract

The MtrCDE multidrug pump, from *Neisseria gonorrhoeae*, is assembled from the inner and outer membrane proteins MtrD and MtrE, which are connected by the periplasmic membrane fusion protein MtrC. Although it is clear that MtrD delivers drugs to the channel of MtrE, it remains unclear how drug delivery and channel opening are connected. We used a vancomycin sensitivity assay to test for opening of the MtrE channel. Cells expressing MtrE or MtrE-E434K were insensitive to vancomycin; but became moderately and highly sensitive to vancomycin respectively, when coexpressed with MtrC, suggesting that the MtrE channel opening requires MtrC binding and is energy-independent. Cells expressing wild-type MtrD, in an MtrCE background, were vancomycin-insensitive, but moderately sensitive in an MtrCE-E434K background. The mutation of residues involved in proton translocation inactivated MtrD and abolished drug efflux, rendered both MtrE and MtrE-E434K vancomycin-insensitive; imply that the pump–component interactions are preserved, and that the complex is stable in the absence of proton flux, thus sealing the open end of MtrE. Following the energy-dependent dissociation of the tripartite complex, the MtrE channel is able to reseal, while MtrE-E434K is unable to do so, resulting in the vancomycin-sensitive phenotype. Thus, our findings suggest that opening of the OMP via interaction with the MFP is energy-independent, while both drug export and complex dissociation require active proton flux.

## Introduction

Gram-negative bacteria utilize homologous tripartite-transport systems to pump cytotoxic compounds, including antibiotics and metals (Pietras *et al*., 2008; Blair and Piddock, 2009; Misra and Bavro, 2009; Su *et al*., 2011a) from the cell. These tripartite pumps are composed of inner (IMP) and outer (OMP) membrane proteins, which are connected by a periplasmic membrane fusion protein (MFP). There are 3D structures for all of the individual components: for example, in the case of the AcrABTolC multidrug pump and CusABC copper pump from *Escherichia coli*, the structure of the IMPs AcrB (Murakami *et al*., 2002; Yu *et al*., 2003) and CusA (Long *et al*., 2010), and of their cognate MFPs, AcrA (Mikolosko *et al*., 2006) and CusB (Su *et al*., 2009), and OMPs, TolC (Koronakis *et al*., 2000) and CusC (Kulathila *et al*., 2011), have been determined. While the different tripartite pumps use structurally related MFPs and OMPs they use at least three different types of IMPs: predominantly proton-driven transporters, belonging to the RND and MF family, but some also use ABC transporters. For example, the AcrABTolC multidrug pump and CusABC copper pump utilize the RND transporters AcrB (Murakami *et al*., 2002) and CusA (Long *et al*., 2010), respectively, while the *E. coli* EmrABTolC and MacABTolC antibiotic pumps utilizes the MF and ABC transporters EmrB (Lomovskaya and Lewis, 1992) and MacB (Kobayashi *et al*., 2001) respectively. In addition, the OMPs of the TolC family also participate in Type I secretion systems for protein toxins, such as the HlyBDTolC pump that secretes α-haemolysin (HlyA) (Holland *et al*., 2005).

IMPs belonging to the RND family, represented by AcrB and CusA, as well as the OMPs, such as TolC and CusC, function as trimeric assemblies. There is evidence that the periplasmic domains of AcrB and TolC contact one another, while AcrA stabilizes this interaction by binding across these proteins (Tikhonova and Zgurskaya, 2004; Touzé *et al*., 2004; Tamura *et al*., 2005). Cross-linking studies indicate that the α-helical coiled-coil hairpin of AcrA fits into intra-protomer grooves of the open state of TolC, while its β-/lipoyl domains interact with the periplasmic domain of AcrB, at the interface between adjacent subunits (Lobedanz *et al*., 2007; Symmons *et al*., 2009). However, recent studies indicate that AcrA has a propensity to form dimers, which can then assemble into hexamers (Tikhonova *et al*., 2011; Xu *et al*., 2011). These findings have led to two different models for the assembly: one in which the AcrA dimers bind to a pair of sites, presumably the intra- and inter-protomer groves, on the surface of TolC and AcrB, strengthening their interaction with one another (Tikhonova *et al*., 2011); while in the other, the dimers form a hexameric channel, interconnecting AcrB to TolC (Xu *et al*., 2011). Our previous studies of the MtrCDE pump from *Neisseria gonorrhoeae* also revealed that the MFP MtrC forms dimers that assemble into a hexameric channel, which has an aperture sufficient to accommodate the MtrE trimer (Janganan *et al*., 2011a,b). Cross-linking the MtrC dimers in the presence of MtrE stabilized an MtrC : MtrE assembly with a stoichiometry of 6:3. However, we found that the α-helical hairpin domain of MtrC could be cross-linked to the α-helical domain of MtrE, consistent with it binding to intra- and inter-protomer grooves on the surface of trimeric MtrE (Janganan *et al*., 2011b). Since we also found that trimeric MtrD interacts with trimeric MtrE, albeit weakly, our findings indicate that the MtrCDE pump is assembled with an MtrD : MtrC : MtrE stoichiometry of 3:6:3, in which MtrD and MtrE are held together by MtrC binding across their surfaces. Importantly, recent crystallographic studies of the CusAB complex also revealed an assembly with a stoichiometry of 6:3 (Su *et al*., 2011b).

Despite the wealth of structural data some fundamental questions on the function of bacterial tripartite multidrug pumps remain unanswered, in particular the role of the membrane fusion proteins in energy transduction between the inner and outer membrane proteins. Previously we isolated a mutant of *mtrE*, encoding an E434K derivative, which exhibits a higher propensity of opening; however, it only does so in response to the binding of MtrC (Janganan *et al*., 2011a). Consistent with MtrC binding to the intra- and inter-protomer grooves of MtrE, mutations in these grooves hindered pump function. It was possible to introduce cysteines into the MtrE grooves and the MtrC hairpin that could be cross-linked, locking the pump in the closed-channel state (Janganan *et al*., 2011b). However, there remains the fundamental question of the role of MtrD in controlling the activity of the pump. Herein, we present evidence that a non-functional MtrD, with charged residues in the trans-membrane (TM) helices mutated to inhibit proton translocation, blocks the MtrE channel because it fails to dissociate from the tripartite complex. This is the first report that the pumping of drugs into the channel of the OMP and its dissociation from the IMP both require energy derived from proton translocation through the IMP.

## Results

### Full-length MtrC is required to induce opening of the MtrE channel

**Fig. 1:**
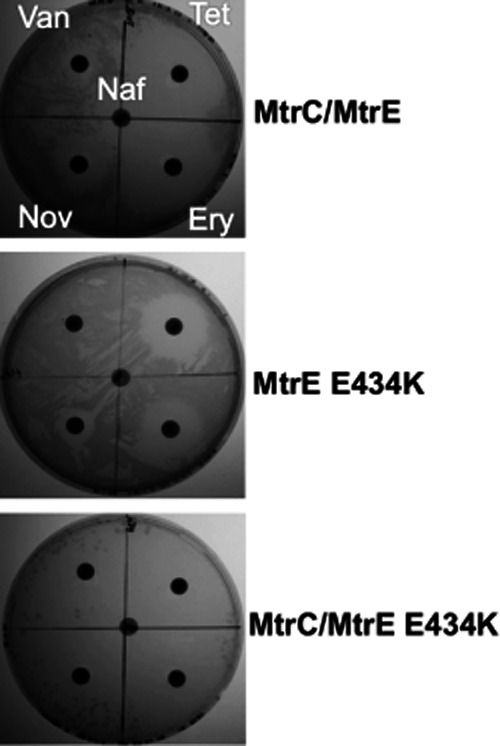
An E434K derivative of MtrE can be used to detect channel opening. Disc-diffusion assays were used to determine if KAM3(DE3) *E. coli* cells expressing *mtrCDE* genes and mutants were susceptible to vancomycin, which can be used as a marker of whether the OMP MtrE is open or closed, since it is too large to readily diffuse across the OM but can enter cells via an open OMP. A set of plates showing the disc-diffusion assays for cells expressing (i) MtrC and MtrE, (ii) MtrE E434K and (iii) MtrC and MtrE E434K. It is notable that cells expressing MtrE E434K alone were insensitive to vancomycin, MtrC/MtrE were moderately sensitive to vancomycin, and MtrC/MtrE E434K failed to grow in the presence of vancomycin. Disc-diffusion assays were performed in triplicate, generally assaying for growth around discs impregnated with the following antibiotics: vancomycin (Van; 30 μg), nafcillin (Naf; 1 μg), tetracycline (Tet; 30 μg), erythromycin (Ery; 10 μg) and novobiocin (Nov; 5 μg).

The MtrE open-state channel can be detected by determining the susceptibility of cells to vancomycin, which only enters cells by passive diffusion very slowly due to its large size, but can gain entry readily through an open OMP (Bavro *et al*., 2008; Janganan *et al*., 2011a,b). Cells expressing MtrE or its E434K derivative are insensitive to vancomycin; however, when expressed with MtrC, the cells expressing MtrE become moderately susceptible to vancomycin, while those expressing MtrE E434K become highly susceptible to (discs impregnated with 30 μg of) vancomycin and fail to grow (Fig. 1; Table 1). This behaviour indicates that MtrC induces opening of the MtrE channel and presumably stabilizes the fully open-channel state of MtrE E434K. Previously we established that the purified N-terminal α-helical hairpin domain, incorporating residues 103–183, of MtrC, retains α-helical structure and binds to MtrE and its E434K derivative (Janganan *et al*., 2011a,b). Indeed, isothermal microcalorimetry (ITC) studies revealed that while NT-MtrC binds to MtrE with a *K*_d_ of 9.8 μM, the hairpin binds to MtrE and MtrE E434K with a *K*_d_ of 2.2 μM and 0.13 μM respectively (Janganan *et al*., 2011a,b). Considering that MtrE binds the hairpin with relatively high affinity, we sought to determine if binding of the hairpin would be sufficient to induce opening of the MtrE channel. To test whether the hairpin could induce opening of the MtrE channel, we expressed it with a gIII signal sequence that targeted it to the periplasm (see *Experimental procedures*). Cells expressing the hairpin with MtrE or MtrE E434K were insensitive to vancomycin, indicating that it is incapable of inducing channel opening (Table 1). As a control, we tested and found that cells expressing NT-MtrC, targeted to the periplasm with a gIII signal sequence, were sensitive to vancomycin and failed to grow (Table 1). Thus, suggesting that the lipoyl, β-barrel and/or MP domains of MtrC, which interact with MtrD, relay conformational changes in MtrD to MtrE, inducing opening of its channel. Considering this behaviour we also tested the effect of expressing MtrD with MtrCE and MtrCE E434K, revealing that the cells were, insensitive and only moderately sensitive to (discs impregnated with 30 μg of) vancomycin respectively (Table 1). Furthermore, strains expressing pACYC-mtrCDE, and control strains with the pACYC vector, had an MIC of 512 μg ml^−1^, while cells expressing pACYC-mtrCDE-E434K and pACYC-mtrCE-E434K had MICs of 64 μg ml^−1^ and 16 μg ml^−1^ respectively (Janganan *et al*., 2011a). It is not surprising then that cells expressing *mtrCDE* grew without any apparent inhibition around a disc impregnated with 30 μg of vancomycin, while the cells expressing *mtrCDE434K* had a moderate zone of inhibition, and those expressing *mtrCE434K* failed to grow to any extent (Table 1). When compared with the susceptibility of cells expressing MtrCE and MtrCE E434K to vancomycin, these data indicate that MtrD retards vancomycin entry, presumably by either re-closing the MtrE channel and/or the occlusion of the periplasmic end of MtrE during complex formation.

**Table 1:** The effect of the MtrC hairpin on the vancomycin resistance of *Escherichia coli* cells expressing MtrE

Strain	Vancomycin sensitivity (mm)
MtrE	NI
MtrE/MtrC	9 ± 1
MtrE E434K	NI
MtrE E434K/MtrC	NG
MtrE E434K/gIII-NT-MtrC	NG
MtrE/gIII-Hairpin	NI
MtrE E434K/gIII-Hairpin	NI
MtrC/MtrD/MtrE	NI
MtrC/MtrD/MtrE E434K	10 ± 1

The indicated MtrE proteins were expressed from pACYC (and pET21a) and the MtrC and hairpin, with an N-terminal gIII signal sequence, proteins from pET24a in KAM3(DE3) *E. coli* cells in the presence of 30 μg of vancomycin. The inhibitory zone diameter is given in mm. All disc-diffusion assays were undertaken in triplicate and the average result given. Abbreviations: NI, no inhibition of growth; NG, no growth.

**Fig. 2:**
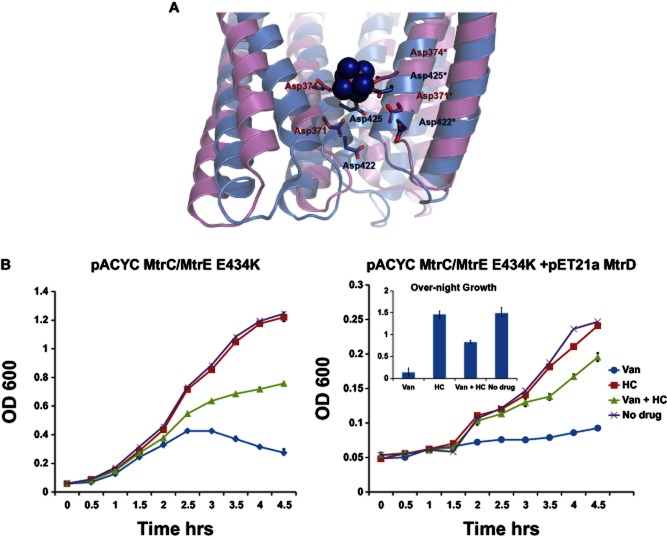
Vancomycin entry into cells through MtrE is blocked by hexamminecobalt. A. A homology model of the MtrE channel based upon a comparative sequence analysis of MtrE and TolC. This model demonstrates that Asp371 and Asp374 in TolC, which form a binding site for hexamminecobalt (III) chloride (HC), are conserved in MtrE and exposed to the internal channel cavity, thus likely forming a similar arrangement to that in TolC. The MtrE model is in blue; two subunits of the TolC–HC complex (1TQQ.pdb) are in magenta; HC is in spacefill; residues Asp371 and Asp374 from TolC, and the homologous Asp422 and Asp425 from MtrE, are shown as sticks. B. A series of curves showing the relative growth, measured as the OD_600_, for *E. coli* cells C43(Δ*acrB*) transformed with pACYC-MtrC-MtrE(E434K) (left set of graphs) and pACYC-MtrC-MtrE(E434K) and pET21a-MtrD (right set of graphs). Cells were grown in the absence of drugs (×); or in the presence of HC (▪, 25 μM), vancomycin (♦, 6.4 μg ml^−1^), of HC and vancomycin (▴). Each bar is the average of at least three measurements, with error bars representing the standard deviation of these measurements from the average. Cells transformed with pACYC and pET grew slower than those transformed with pACYC only; but, as shown in the bar chart, attained a comparable OD after overnight growth.

We sought to provide further evidence that vancomycin enters the cells through open MtrE. Large positively charged ions, such as hexamminecobalt (III) chloride (HC), have been shown to block the TolC channel via co-ordination with the ‘second selectivity’ gate residues Asp371 and Asp374, which form two concentric circles of negative charges in the inner cavity of TolC (Andersen *et al*., 2002b). Indeed TolC has been successfully crystallized in complex with HC (Higgings *et al*., 2004) and the binding has been shown to block the channel in electrophysiological studies (Andersen *et al*., 2002a,b). The affinity of the binding has been estimated to be in the range ∼ 20 nM from ITC measurements (Higgins *et al*., 2004). The TolC channel is effectively sealed for transport unless the Asp-ring is broken, which is suggested to be one of the roles of the MFP (Bavro *et al*., 2008). Hexammine cobalt stabilizes the Asp-ring, and hence renders the channel non-conductive to larger cargo. Using comparative sequence analysis and homology modelling studies of the MtrE channel (Janganan *et al*., 2011a), here we demonstrate that the Asp371 and Asp374 are conserved in MtrE, and the corresponding residues (Asp422 and Asp425 respectively) are exposed to the internal channel cavity, thus likely forming a similar arrangement to that in TolC (Fig. 2A). Hence, we capitalized on this to test whether blockage of the MtrE channel with HC would also result in a lowered sensitivity to vancomycin, as the latter is using the channel for cell entry. A growth curve analysis, using a series of increasing HC concentrations, revealed that concentrations above 50 μM were inhibitory for cell growth: in subsequent experiments 25 μM HC was used, which did not significantly inhibit cell growth (Fig. 2B). Consistent with HC blocking the MtrE channel, 25 μM HC caused a clear and significant decrease in the sensitivity of the cells, expressing both MtrC along with MtrE E434K, to 6.4 μg ml^−1^ vancomycin (Fig. 2B; left graph). Indeed, we found that the inclusion of 25 μM hexammine cobalt caused an increase in the MIC for vancomycin for cells expressing MtrCE E434K from 16 μg ml^−1^ to 64 μg ml^−1^. It was notable that HC appeared more efficient at blocking the entry of vancomycin into cells expressing MtrC and MtrE E434K with MtrD (Fig. 2B; right graph and bar chart). The vancomycin MIC for these cells increased from 64 μg ml^−1^ to 256 μg ml^−1^ when treated with 25 μM hexamine cobalt. Presumably, because both HC and MtrD can block the periplasmic end of the MtrE channel, they work together to more effectively block vancomycin entry into the cells.

**Fig. 3:**
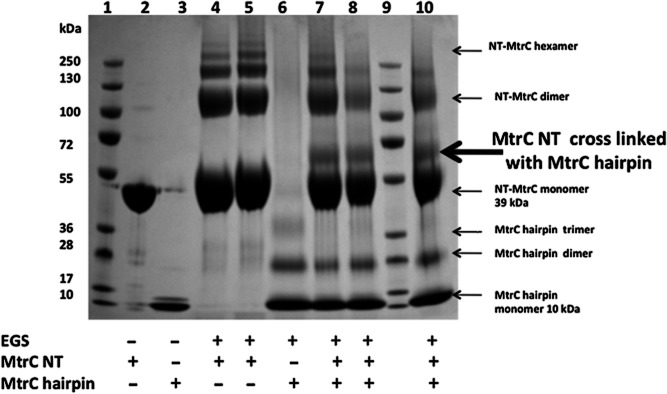
The hairpin of MtrC forms heterodimers with MtrC. To test for heterodimers of the MtrC hairpin with NT-MtrC, (1 mM) EGS was added to (8 μM) MtrC hairpin mixed with (8 μM) NT-MtrC (lanes 7, 8 and 10) and analysed by SDS-PAGE. The control lanes are as follows: Mr-standard (lanes 1 and 9), NT-MtrC (lane 2), hairpin (lane 3), NT-MtrC + EGS (lanes 4 and 5) and hairpin + EGS (lane 6). An arrow indicates the bands corresponding to the formation of heterodimers of hairpin/NT-MtrC.

Considering that the MtrC hairpin did not induce opening of the channel of MtrE suggested that it might have the potential to interfere with the assembly of the pump by preventing the binding of full-length MtrC to MtrE. However, we found that cells expressing MtrCDE with the hairpin consistently grew better than those expressing MtrCDE alone, in the presence of antibiotics; while cells expressing the hairpin either alone, or with MtrD, behaved as the control cells, which were sensitive to antibiotics and grew poorly (Fig. S1). Furthermore, we found that for the cells expressing the hairpin there was a significant decrease in their susceptibility to a range of antibiotics, and the cells were insensitive to vancomycin (Table 2). These data clearly indicate that the hairpin interacts with the pump, apparently activating it, rather than preventing its assembly. Providing a plausible explanation for this effect, chemical cross-linking, with EGS, revealed that the hairpin forms heterologous dimers with NT-MtrC (Fig. 3). Considering that there is mounting evidence that the MFP forms dimers (Janganan *et al*., 2011b; Tikhonova *et al*., 2011; Xu *et al*., 2011) that interact with three pairs of intra- and inter-protomer grooves on the surface of the surface of the trimeric OMP (Janganan *et al*., 2011b; Ferrandez *et al*., 2012), our data can be rationalized if the MFP binds to one of these grooves, while the hairpin bindings to the other, to activate the pump. Indeed it is expected that the two MFP-binding grooves on the surface of the OMP will represent two different, degenerate binding interfaces with different affinities, something that has also been previously suggested by ITC and SPR binding assays (Janganan *et al*., 2011a,b; Tikhonova *et al*., 2011). It is possible that while full-length MtrC is needed to initiate opening of the MtrE channel, the hairpin is capable of stabilizing the already open, activated state of the channel, but it is clearly not capable of conveying the opening on its own. Such data also agrees with the idea of the OMP having two different affinity sites that engage MFPs in a different fashion (Janganan *et al*., 2011b; Tikhonova *et al*., 2011).

**Table 2:** The effect of the MtrC hairpin on the antibiotic susceptibility of *Escherichia coli* cells expressing components of the MtrCDE multidrug pump

Constructs	Naf 1 μg	Tet 30 μg	Ery 10 μg	Nov 5 μg	Van 30 μg
pACYC-MtrCDE + pET24a	13 ± 1	26 ± 1	21 ± 1	20 ± 1	NI
pACYC-MtrCDE + pET24a-gIII MtrC hairpin	9 ± 1	22 ± 1	18 ± 1	19 ± 2	NI
pACYC + pET24a-gIII MtrC hairpin	20 ± 1	36 ± 1	30 ± 0.1	20 ± 1	NI
pACYC-MtrE	15 ± 1	33 ± 3	27 ± 2	19 ± 1	NI
pACYC-MtrE + pET24a-gIII MtrC hairpin	15 ± 1	32 ± 1	26 ± 1	19 ± 1	NI
pACYC MtrD	16 ± 1	39 ± 3	30 ± 1	19 ± 2	NI
pACYC MtrD + pET24a-gIII MtrC hairpin	15 ± 1	40 ± 2	30 ± 1	20 + 1	NI
pACYC Vector + pET24a	18 ± 2	35 ± 2	32 ± 3	21 ± 1	NI

The indicated Mtr proteins were expressed from pACYC and the MtrC hairpin, with an N-terminal gIII signal sequence, from pET24a in KAM3(DE3) *E. coli* cells. The inhibitory zone diameter is given in mm. All disc-diffusion assays were undertaken in triplicate and the average result given. The amount of antibiotic impregnating each disc is given. Abbreviations: NI, no inhibition of growth; Naf, nafcillin; Tet, tetracycline; Ery, erythromycin; Nov, novobiocin; Van, vancomycin.

### The dissociation of MtrD from MtrE requires energy that is provided by proton translocation

Since our studies revealed that the C-terminal domain of MtrC, which interacts with MtrD, is required to facilitate opening of the MtrE channel, this suggested that it might play a role in conveying conformational changes from MtrD to MtrE. If this were the case, then presumably MtrD would need to be functional to facilitate the opening of MtrE. We sought to investigate this possibility by inactivating MtrD and determining if it could support the opening of MtrE. Previous studies of other RND pumps have revealed three residues (e.g. D407 and D408 in TMH 4, and K939 in TMH 10, of MexB; and the homologous D407, D408 and K939 residues of AcrB) that are essential and proposed to function as part of the proton-relay system (Guan and Nakae, 2001; Takatsuka and Nikaido, 2006; Seeger *et al*., 2009). Bioinformatic analysis indicates that the corresponding residues, D405, D406 and K948, are conserved in MtrD (Fig. S2), based on the close homology of MtrD with both AcrB from *E. coli* (48.3% identity) and MexB from *Pseudomonas* (48.6% identity) respectively. The secondary structural elements appear well preserved within the group as judged by ALIGN (Cohen, 1997).

**Fig. 4:**
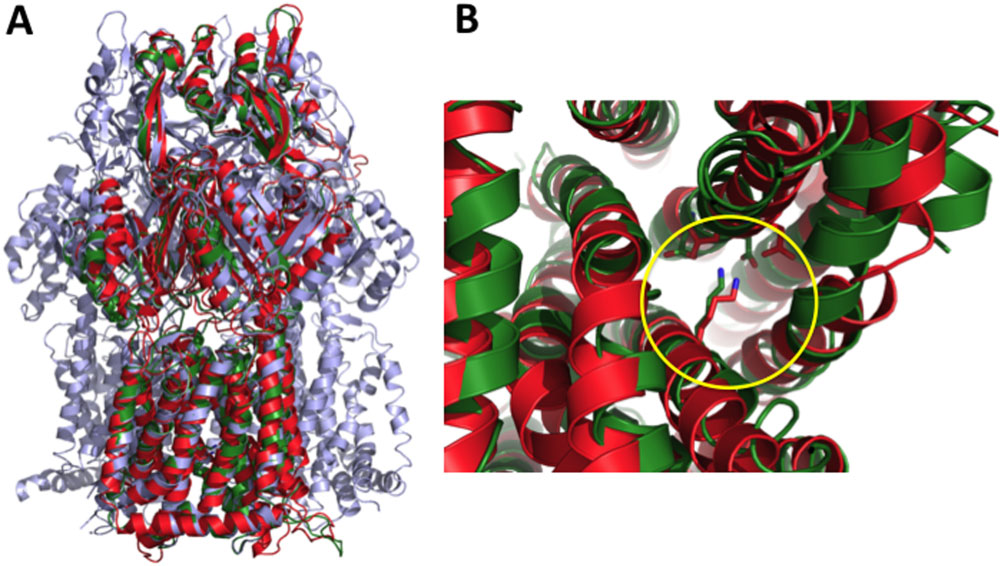
A homology model for MtrD. A. An overlay of the MtrD models with AcrB trimer. The MexB-based model is in green; the AcrB-based model is in red; and the AcrB 2DHH structure is shown as a light blue trimer. This figure was generated using Pymol. B. This homology modelling allows for an unambiguous location of the residues in the TM helices, and as can be seen, D405, D406 and K948 (highlighted by a yellow circle) appear within bonding distance in both models. In combination with our biochemical data, this provides very strong support to the idea of conservation of this charged network across different RND transporters and the clearly indicates that these residues perform in a similar fashion to these previously established in AcrB.

Accordingly, we chose these residues for mutagenesis, with the aim of inactivating the pump. All of these residues were found to be essential for MtrCDE conferred resistance to antibiotics (Table 3). Furthermore, none of the double or triple mutants appeared more sensitive than any of the single mutants suggesting that they have a concerted function, presumably in relaying protons across the membrane (Eicher *et al*., 2009; Pos, 2009; Seeger *et al*., 2009). To further confirm the hypothesis that these residues could play a role of a proton-relay triad and are colocalized in the MtrD, and taking advantage of the number of high-resolution structures that are available for these proteins, we generated two homology models of MtrD, based on 2DHH.pdb (AcrB; Murakami *et al*., 2002) and 2V50.pdb (MexB; Sennhauser *et al*., 2009) structures respectively. When using the SSM superposition algorithm of COOT (Emsley and Cowtan, 2004), the two independent models align with a core RMSD of 2.3 Å, mainly due to a few flexible loops and hairpins, and are overall highly convergent, with the TM part in particular having an RMSD of less than 1 Å (Fig. 4A). As can be seen in Fig. 4A, in both models the residues are in close proximity and likely interacting with each other. This allows for an unambiguous location of the residues in the TM helices and, as can be seen in Fig. 4B, the D405, D406 and K948 appear within bonding distance in both models. In combination with our biochemical data, this provides very strong support to the idea of conservation of this charged network across different RND transporters and suggests that these residues perform in a similar fashion to those previously established in AcrB.

**Table 3:** Identification of MtrD mutations that impair the function of the MtrCDE pump

Constructs	Tet 30 μg	Ery 10 μg	Nov 5 μg	Van 30 μg
pACYC MtrCE + pET21a MtrD WT	29 ± 2	20 ± 1	12 ± 2	NI
pACYC MtrCE + pET21a MtrD D405K	40 ± 1	30 ± 1	17 ± 1	NI
pACYC MtrCE + pET21a MtrD D406K	40 ± 1	27 ± 2	18 ± 1	NI
pACYC MtrCE + pET21a MtrD K948E	40 ± 1	27 ± 2	19 ± 1	NI
pACYC MtrCE + pET21a MtrD D405K/D406K	40 ± 1	30 ± 1	18 ± 1	NI
pACYC MtrCE + pET21a MtrD D405K/D406K/K948E	40 ± 2	30 ± 2	19 ± 1	NI
pACYC + pET21a	38 ± 1	29 ± 1	17 ± 1	NI

The MtrCE proteins were expressed from pACYC and the MtrD derivatives from pET21a in KAM3(DE3) *E. coli* cells. The inhibitory zone diameter is given in mm. All disc-diffusion assays were undertaken in triplicate and the average result given. The amount of antibiotic impregnating each disc is given. Abbreviations: NI, no inhibition of growth; WT, wild-type; Tet, tetracycline; Ery, erythromycin; Nov, novobiocin; Van, vancomycin.

**Fig. 5:**
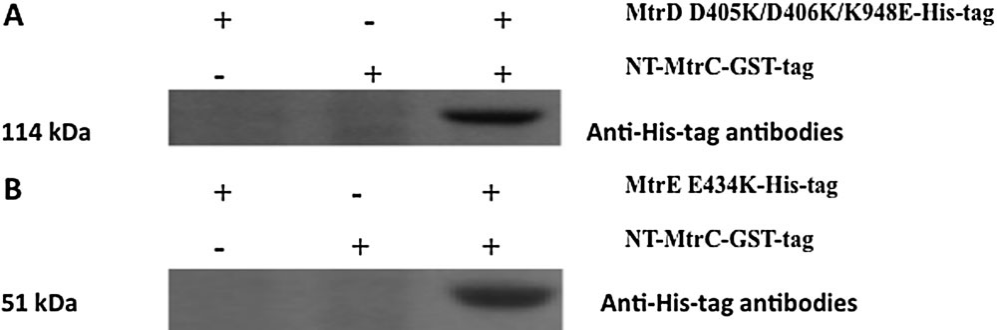
MtrC binds to both MtrE E434K and MtrD D405K/D406K/K948E. The purified NT-MtrC GST-tagged protein was immobilized on GST column beads, to which was added purified His-tagged MtrE E434K (B) or MtrD D405K/D406/K948E (A) prey protein. The beads were washed with Tris buffer, containing 0.1% w/v βDDM, then boiled at 90°C for 5 min to elute the complex. Western blotting, using antibodies to the His-tag, was used to detect the MtrE and MtrD prey proteins. As negative controls, either the bait protein or the prey protein was omitted from the assay (the presence and absence of proteins is indicated by ‘+’ and ‘−’). As shown in each assay when either of these proteins was omitted from the assay, the corresponding prey protein could not be detected. These assays indicate that MtrC interacts with MtrE E434K and MtrD D405A/D406A/K948K.

In common with cells expressing wild-type MtrCDE, all the *mtrD* mutants were insensitive to (discs impregnated with 30 μg of) vancomycin (Table 4), and had an MIC of 512 μg ml^−1^, indicating that the MtrE channel is closed or blocked. However, this behaviour could arise if, for the *mtrD* mutants, MtrD became uncoupled from MtrCE. To test for this possibility, we coexpressed the *mtrD* mutants with *mtrC*/*mtrE* E434K. As noted above, while cells expressing MtrC/MtrE E434K are sensitive to vancomycin and fail to grow, we found that cells expressing MtrC/MtrD/MtrE E434K are moderately sensitive (Table 4). If MtrD was decoupled from MtrE E434K, in the cells expressing the *mtrD* mutants with *mtrC*/*mtrE* E434K, then they should be highly sensitive to vancomycin and fail to grow. Importantly, the cells can only become resistant to vancomycin if they produce MtrD that can interact with MtrC/E E434K to prevent MtrC unlocking the MtrE E434K channel. Indeed, if MtrD failed to express at all, then the cells would only produce MtrC/MtrE E434K, and would fail to grow due to their sensitivity to vancomycin. Our analysis revealed that cells expressing MtrD D405K, D406K and/or K948E with MtrC/MtrE E434K were insensitive to (discs impregnated with 30 μg of) vancomycin (Table 4), and had an MIC of 512 μg ml^−1^. However, as expected, the mutants were sensitive to a range of antibiotics that are substrates of the pump (Table 4). Furthermore, pull-down assays revealed that MtrC interacts with both MtrE E434K and MtrD D405K/D406K/K948E, indicating that the mutations in these proteins did not impair their ability to interact (Fig. 5). These findings indicate that the MtrD derivatives interact with MtrC and MtrE E434K resulting in a seal of the MtrE channel. Consequently, access to the lumen of the MtrE channel requires functional MtrD. Considering that the D405K, D406K and K948E derivatives of MtrD are most likely compromised in their ability to translocate protons, suggests that proton translocation is coupled to exposure of the periplasmic end of the MtrE channel, which is brought about by the dissociation of MtrD from MtrE.

**Table 4:** MtrD derivatives form a stable complex with MtrE, preventing vancomycin access into the cells

Constructs	Naf 1 μg	Tet 30 μg	Ery 10 μg	Nov 5 μg	Van 30 μg
pACYC MtrCE + pET21a MtrD WT	NI*	29 ± 2	20 ± 1	12 ± 2	NI
pACYC MtrC/E (E434K) + pET21a MtrD WT	NI*	45 ± 1	40 ± 1	21 ± 1	10 ± 1
pACYC MtrC/E(E434K) + pET21a MtrD D405K	NI*	40 ± 1	30 ± 2	20 ± 1	NI
pACYC MtrC/E(E434K) + pET21a MtrD D406K	NI*	39 ± 1	31 ± 1	17 ± 1	NI
pACYC MtrC/E(E434K) + pET21a MtrD K948E	NI*	38 ± 1	28 ± 1	17 ± 1	NI
pACYC MtrC/E(E434K) + pET21a MtrD (D405K/D406K)	NI*	39 ± 2	30 ± 1	20 ± 1	NI
pACYC MtrC/E (E434K) + pET21a MtrD (D405K/D406K/K948E)	NI*	41 ± 1	31 ± 1	20 ± 1	NI
pACYC + pET21a	NI*	38 ± 1	29 ± 2	17 ± 1	NI
pACYC	8 ± 1	36 ± 1	32 ± 1	17 ± 2	NI
pACYC MtrC/MtrE WT	24 ± 1	36 ± 1	37 ± 1	21 ± 1	9 ± 1
pACYC MtrC/MtrE E434K	NG	50 ± 2	40 ± 1	NG	NG

The MtrCE proteins were expressed from pACYC and the MtrD derivatives from pET21a in KAM3(DE3) *E. coli* cells. The inhibitory zone diameter is given in mm. All disc-diffusion assays were undertaken in triplicate and the average result given. The amount of antibiotic impregnating each disc is given. Abbreviations: NI, no inhibition of growth; NI*, pET21a plasmid encoded resistance; WT, wild-type; Naf, nafcillin; Tet, tetracycline; Ery, erythromycin; Nov, novobiocin; Van, vancomycin.

## Discussion

Although our earlier studies provide strong evidence that MtrC plays a role in controlling the activity of the pump by binding to the intra- and inter-protomer grooves of MtrE, what was not clear from such studies was whether binding of the hairpin domain of MtrC is sufficient to induce opening of the MtrE channel. Since our previous *in vitro* studies revealed that the hairpin binds to both MtrE and its E434K derivative (Janganan *et al*., 2011a); herein, we utilized a gIII signal sequence to target the hairpin to the periplasm of cells express MtrE E434K, revealing that the hairpin is unable to unlock the MtrE channel on its own (Table 1). This contrasts with full-length MtrC, or NT-MtrC targeted to the periplasm with a gIII signal sequence, suggesting that the C-terminal domain constrains the conformation of the N-terminal hairpin domain, so that it can productively interact with MtrE E434K to open its channel. The hairpin domain is sandwiched between sequences that contribute residues to the lipoyl, the β-barrel and the membrane proximal (MP) domains, presumably restraining the flexibility of the hairpin (Symmons *et al*., 2009). In the case of AcrA, cross-linking studies have revealed that the α-hairpin domain interacts with TolC (Lobedanz *et al*., 2007), while the other three domains interact with AcrB (Symmons *et al*., 2009). The crystal structure of AcrA (Mikolosko *et al*., 2006), and molecular dynamic simulations (MDS) (Wang *et al*., 2012), revealed that it has large conformational flexibility that largely arises from the relative motions between the α-hairpin and the other domains. Interestingly, a G336C substitution in the MP domain of AcrA inhibited the AcrABTolC pump, indicating that interactions between this domain of AcrA and AcrB are essential for the operation of the pump (Ge *et al*., 2009; Tikhonova *et al*., 2011). Furthermore, while the AcrAB complex cannot interact with a TolC derivative in which the periplasmic end of the channel is open, a complex of AcrB with the G336C derivative of AcrA can interact with this open-state TolC derivative (Tikhonova *et al*., 2011), suggesting that AcrAB interacts with the closed-state TolC and that conformational changes in the MP domain of AcrA are associated with opening of the TolC channel. It seems highly plausible that the C-terminal end, and more precisely the MP domain, of the MFP is necessary for active coupling and energy transduction from the IMP to the OMP.

Active proton translocation by the RND pump would appear to result in the progressive peristaltic motion of the pump (Murakami *et al*., 2006; Seeger *et al*., 2006;2008), which is propagated to the surface of the extrusion protomer (Seeger *et al*., 2008; Nakashima *et al*., 2011; Eicher *et al*., 2012) and hence conceivably communicated to, and resulting in equally dramatic conformational changes, in the MFP. In turn, these conformational changes in the MFP could be used to alter its interaction with the OMP; to induce either opening of the OMP channel or the disengagement of the MFP from the OMP, allowing the IMP/MFP to dissociate from the OMP and hence decoupling of the pump post-drug extrusion. To determine if MtrD communicates with MtrE, we produced a set of functionally impaired *mtrD* mutants and tested the effect of these derivatives on the MtrE channels ability to conduct vancomycin. Cells expressing *mtrC* and *mtrE E434K* along with the non-functional *mtrD* mutants were rendered insensitive to vancomycin, as were cells expressing *mtrE E434K* alone, while cells expressing *mtrC* and *mtrE E434K* were highly sensitive to vancomycin (Table 4). One possibility for this behaviour is that, while MtrC can unlock the closed channel of MtrE E434K, the non-functional MtrD derivatives interact with MtrC, preventing it from unlocking the channel of MtrE E434K. However, since the MtrD derivatives would not be expected to undergo proton-driven conformational changes, it seems unlikely that they could induce the closure of the MtrE E434K channel, which would otherwise be open. An alternative, perhaps more plausible, possibility is that the MtrD derivatives fail to disengage from MtrE E434K, effectively sealing the channel to vancomycin entry. Such an intriguing interpretation would also suggest that channel unlocking by the hairpins of MtrC is energy-independent, while the proton-motive force is used to actively dissociate the complex and re-seal the periplasmic end of MtrE. Considering that cells expressing MtrCDE E434K are sensitive to vancomycin, which can be reversed by the binding of hexamminecobalt to the periplasmic end of MtrE, implies that MtrD can dissociate from MtrE E434K, which remains in the open state; conversely, cells expressing MtrCDE are insensitive to vancomycin, implying that the dissociation of MtrD is concomitant with closure of the MtrE channel. The insensitivity of the cells expressing the non-functional MtrD mutants to vancomycin would be consistent with them failing to dissociate from MtrE E434K. Indeed, in contrast to the substantive changes in the conformation of the periplasmic domain of the wild-type protein, there are only small changes in the conformation of the periplasmic domain of the AcrB D407A, D408A and K940A derivatives (Su *et al*., 2006; Takatsuka and Nikaido, 2006; Nikaido and Takatsuka, 2009); consequently, our MtrD derivatives would not be expected to induce the substantive conformational changes in MtrC that would presumably be necessary to induce its disengagement from MtrE.

**Fig. 6:**
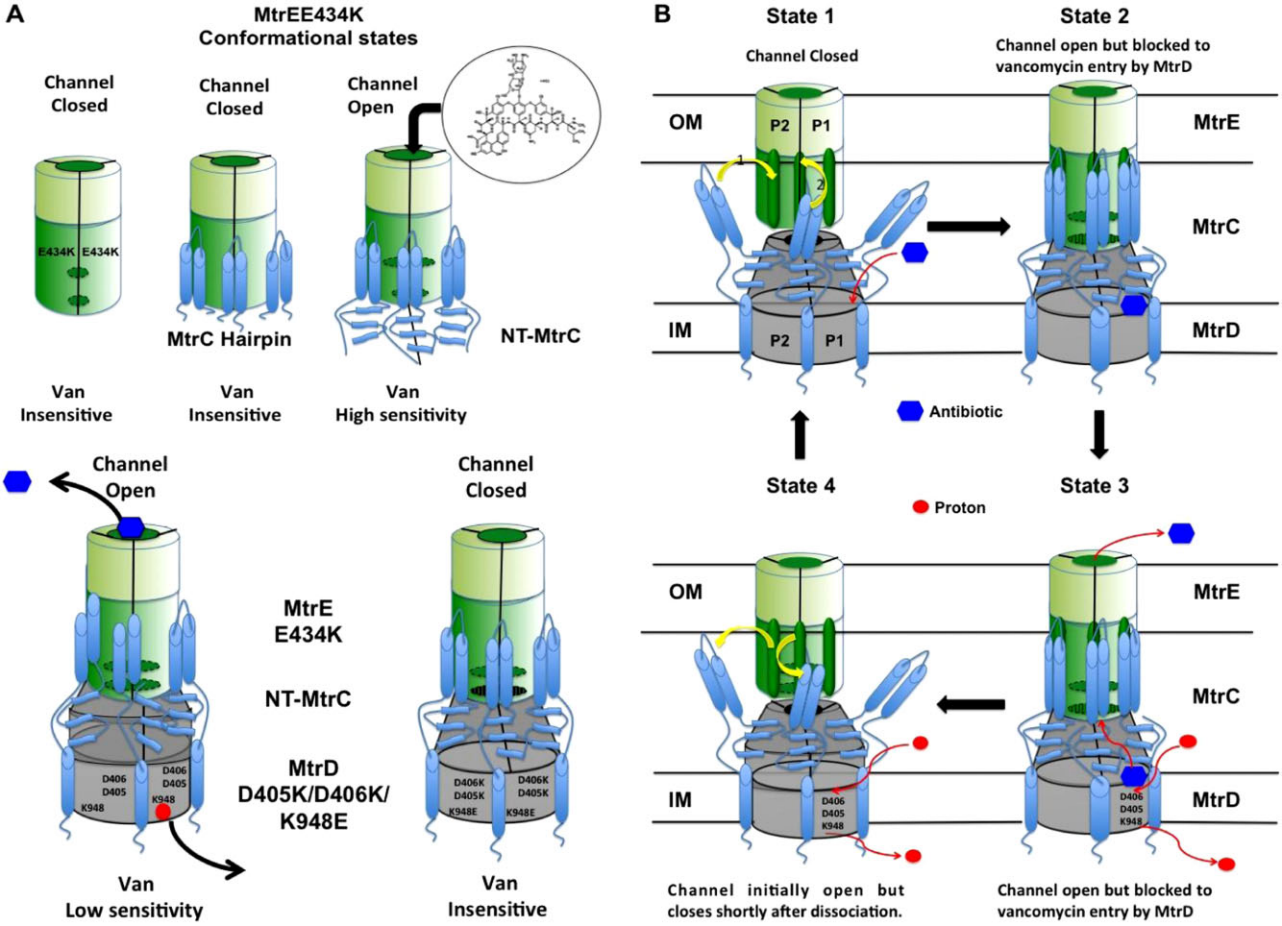
A schematic model for the assembly and function of the MtrCDE tripartite pump. A. Additional conformational states for assemblies of the components of the MtrCDE tripartite pump identified using the E434K, vancomycin leaky, derivative of MtrE. In the case of the MtrE E434K derivative, both the primary and the secondary gates of the channel are open, so that it can leak vancomycin. However, MtrD is capable of sealing the channel, to prevent vancomycin entry, until it dissociates from MtrE E434K. B. A schematic model for the action of the MtrCDE pump. MtrC and MtrD form a complex (state 1) that in response to drug binding interacts with MtrE (state 2). The binding of MtrC to the intra and inter-protomers grooves, and then of MtrD, to the periplasmic tip, of MtrE opens the secondary and primary gates, respectively, of the MtrE channel. The binding of protons to the membrane domain of MtrD induces conformational changes in the periplasmic domain of MtrD that results in transfer of bound drugs to the open MtrE channel (state 3). A further energy-dependent step, possibly involving a second proton translocation event, is then needed to drive the dis-engagement of MtrCD from MtrE (state 4), allowing MtrE to collapse back to its original closed conformation (state 1). The protomers are labelled P1 and P2 (while P3 is hidden from view). The movement pathway of the drug (blue hexagon) and the proton (red circle) is shown as a red curved arrow; while that the sequential binding (marked 1 and 2) of the MtrC hairpin domains to the intra- and inter-protomers grooves of MtrE as yellow curved arrows.

In summary, our studies utilizing MtrE E434K have identified a number of additional conformational states (Fig. 6A), which can be incorporated into a model for the pump mechanism (Fig. 6B). The integral inner membrane protein MtrD, presumably with bound drug, and MtrC form a complex, in which a ring of six MtrC protomers assemble around the MtrD trimer (state 1). This complex can then dock with the MtrE trimer (state 2), which results in opening of the secondary selectivity gate (the aspartate ring) in the MtrE channel, but not the fully open state. The docking of MtrE and MtrD promoted by MtrC leads to final unlocking of the primary selectivity gate of MtrE by the β-hairpins of MtrD, which has already been suggested for TolC-AcrB (Andersen *et al*., 2002c; Tamura *et al*., 2005; Bavro *et al*., 2008). We can only speculate at this stage on the exact sequence of events during the engagement of MtrC with MtrE, but similar to the AcrA/TolC interaction (Bavro *et al*., 2008; Tikhonova *et al*., 2011), sequential engagement of the intra- and inter-protomer grooves is likely. Indeed it seems likely that the inter-protomer groove becomes available only upon the initial binding of the first MtrC monomer in the high-affinity intra-protomer groove, and that this is associated with the disruption of the aspartate gate, while the binding of the second hairpin, to the low-affinity inter-protomer groove, is just to stabilize the open assembly. Conceivably, the second hairpin only engages with MtrE after it has bound to MtrD, to stabilize the MtrE channel in the open conformation. The role of the hairpin in unlocking of the aspartate gate can also be implied by comparative studies of the *Borellia* efflux pump BesABC (Bunikis *et al*., 2008), where the MFP BesA uncharacteristically lacks a hairpin. It is notable however that the corresponding second selectivity gate in the OMP BesC is also not present. The binding of protons to the TM domain of MtrD, and their subsequent passage through the proton relay circuit, composed of the residues D405, D406 and K948, induces conformational changes in the periplasmic domain of MtrD that results in transfer of bound drugs towards the periplasmic end of the MtrE channel which has to be fully open by this stage (state 3). The deep interpenetration of the MFP hairpins with the helical coiled-coils of the TolC family members indicate that this is likely a very stable interaction, which is further confirmed by the fact that this extended binding interface tolerates single mutations and from binding studies that reveal a nanomolar affinity (Touzé *et al*., 2004; Bavro *et al*., 2008; Tikhonova *et al*., 2009; 2011; Janganan *et al*., 2011a,b). Furthermore, these interactions are about an order of magnitude stronger than the corresponding IMP/OMP interactions and are energy-independent (Touzé *et al*., 2004; Janganan *et al*., 2011a; Tikhonova *et al*., 2011). Dissociating such an extended binding interface is likely to require energy, and it is clear that only the IMP can provide this energy. In the absence of energy input, the complex will stay associated permanently, which is supported by our studies that suggest that vancomycin entry is blocked by our inactivated MtrD derivatives, which fail to dissociate from MtrE. Thus, this work provides a tantalizing suggestion that there is at least one more energy-dependent step in the drug expulsion process, and that this is the dissociation of MtrC from MtrE (state 4). Our *in vivo* experiments of the interaction of MtrC with MtrE E434K also clearly imply that MtrC is capable of binding to MtrE in the absence of an energized pump, which also results in channel opening. Thus the OMP–MFP interaction appears to be energy-independent and likely results in a low-energy state that is stabilized by the extensive helical coiled-coils interfaces. Although it is clear that MtrD must provide the energy for dissociation of the MtrCDE tripartite complex, it is not known whether this occurs concomitantly with drug extrusion. Once MtrC is removed from MtrE, MtrD will be able to dissociate from MtrE, and the outermost selectivity gate of MtrE will spontaneously collapse, while MtrC and MtrD may remain associated together for another drug cycle. It seems plausible that the proton-driven conformational changes in MtrD are coupled to conformational changes in MtrC that trigger its dissociation from MtrE.

## Experimental procedures

### Strains and plasmids

The *E. coli* strains and plasmids used are described in Table S1, and the primers used for construction of plasmid vectors in Table S2. For *in vivo* studies of the effects of MtrC and its hairpin upon MtrE and its E434 derivative, wild-type and mutant *mtrE* were expressed from pACYC Duet, while NT-*mtrC* and its *hairpin*, both bearing a gIII signal sequence to target the proteins to the periplasm, were expressed from pET24a. These experiments were also repeated using (i) pACYC Duet to express both *mtrE* and *mtrC*/*gIII-hairpin*, and (ii) pET21a and pET24a to express *mtrE* and *mtrC/gIII-hairpin* respectively. Such studies confirmed that the resulting effects were independent of the vector pair used for coexpression of these proteins (data not shown). For *in vivo* studies of the effect of introducing mutations into *mtrD*, the pET21a-mtrD plasmid was used as the template for site-directed mutagenesis using a QuikChange XL kit from Stratagene, with the primers given in Table S3.

### Protein overexpression and purification

The MtrD, MtrE, NT-MtrC and MtrC hairpin proteins were purified as fusion proteins with a six-histidine tag, while GST-tagged NT-MtrC was used for pull-down assays. *E. coli* cells [strain BL21(DE3) transformed with the relevant pET21a vector for expression MtrD, MtrE, NT-MtrC and MtrC hairpin proteins] were grown at 37°C in 2× YT media, with shaking at 220 r.p.m., until the cells density reached an *A*_600_ of 0.6, when 0.5 mM IPTG was added, the temperature adjusted to 25°C and cells left to overexpress proteins for 6 h for membrane proteins and 3 h for soluble proteins. Cells were harvested by centrifugation at 4°C and resuspended in 20 mM Tris-HCl buffer, 300 mM NaCl, pH 7.5, 10% v/v glycerol. Complete protease inhibitor (Roche) and DNase I (Sigma) was added to the cell paste before lysing the cells, via three passes (e.g. 1 × 10 kpsi and 2 × 25 kpsi) using a (Z-plus 1.1 kW) Constant Systems cell disrupter. Cell debris was removed by centrifugation at 37K *g* (45 min at 4°C), and cell membranes isolated by ultracentrifugation at 125K *g* (90 min at 4°C) for membrane proteins, while the supernatant was collected for soluble proteins. Membranes pellets were then solubilized in 20 mM Tris-HCl buffer, pH 7.5, 100 mM NaCl, 10% (v/v) glycerol, 2% w/v β-d-dodecyl maltoside (βDDM) (Anatrace). For the purification of MtrD and MtrE proteins the membranes pellets were dissolved in 2% w/v βDDM and the protein were purified under 0.1% w/v βDDM by affinity chromatography using a Ni^2+^-charged HiTrap chelating column (GE Healthcare). After desalting, samples were subjected to ion exchange chromatography, using either a (GE Healthcare) HiTrap Q or a SP column, for the MtrD and MtrE proteins respectively, which were eluted using a NaCl gradient. For the purification of His-tagged Δ1–34-MtrC (NT-MtrC) and 100–183 α-helical hairpin of MtrC, the supernatant was purified by affinity chromatography using a HiTrap Ni^2+^-chelating column, followed by gel filtration chromatography, using a 120 ml Hiload 16/600 Superdex 200 prep grade column, in 20 mM Tris-HCl buffer, pH 7.5, 300 mM NaCl, 10% v/v glycerol. In addition, we found that the hairpin could be purified from cells that had been subjected to an osmotic shock to release periplasmic proteins: confirming the presence of the hairpin in the periplasm. For the purification of GST-tagged NT-MtrC, the protein was overexpressed by growing the *E. coli* cells [strain BL21(DE3) transformed with pGEX6p-3-NT-MtrC] at 25°C overnight with 0.2 mM IPTG. After cell lysis (in PBS) and centrifugation at 37K *g* (30 min at 4°C), the supernatant was mixed with GST resin and incubated at 4°C for 30 min. Then the resin was packed into a gravity flow column, washed with PBS and finally eluted with 10 mM glutathione (in PBS). The eluted protein was concentrated (to give a volume of about 3 ml) using a 50 kDa cut-off Vivaspin concentrator, and loaded onto a gel filtration column (Hiload 16/600 Superdex 200) for further purification.

### Disc diffusion assays

*Escherichia coli* cells [strain KAM3(DE3) (Mima *et al*., 2007)] were grown to an OD_600_ of about 0.5, induced with 0.1 mM IPTG, the cells were diluted to an OD_600_ of 0.3 and then 350 μl of cell culture was spread on an MHA plate previously incorporated with 0.1–0.2 mM IPTG; antibiotic discs were placed on the plate medium and incubated at 37°C overnight. The zone of culture growth inhibition was determined. The discs used were impregnated with 1 μg of nafcillin, 30 μg of tetracycline, 10 μg of erythromycin, 5 μg of novobiocin and 30 μg of vancomycin. Western blotting with protein sequence-specific antibodies and/or antibodies to the His-tag was routinely used to test the cells used for antibiotic sensitivity assays for the expression of the MtrC, MtrD and MtrE proteins. None of the mutants used in our studies had protein levels substantively different than found in cells expressing the wild-type proteins; and consequently the differences in antibiotic sensitivity are not attributable to differences in the protein expression levels.

### Growth curve assays

*Escherichia coli* cells (Δ*acrB* strains KAM3 or C43) were grown at 37°C in LB, supplemented with 30 μg ml^−1^ chloramphenicol, for selection of pACYC, and/or 100 μg ml^−1^ carbenicillin, for selection of pET, to an OD_600_ of about 0.4, and then induced with 1 mM IPTG for 2–2.5 h at 37°C. The cells were diluted to an OD_600_ of about 0.1, with LB, supplemented with appropriate antibiotics (e.g. 30 μg ml^−1^ chloramphenicol and/or 100 μg ml^−1^ carbenicillin) and for some cultures hexammine cobalt (III) chloride (final concentration 25 μM) and/or vancomycin (final concentration 6.4 μg ml^−1^), and the cells grown at 37°C, while recording their OD every 30 min, until they reached stationary phase. Similarly, after IPTG induction, the cells were diluted with 2× YT media containing antibiotic and monitored for growth after 24 h for MIC measurements.

### Pull-down assays

For pull-down assays, we used the GST-tagged NT-MtrC as the bait protein, to pull-down the His-tagged MtrD and MtrE prey proteins. The purified NT-MtrC GST-tagged protein was immobilized on GST column beads (GE Healthcare), to which was added purified His-tagged MtrE E434K or MtrD D405K/D406/K948E prey protein (5 mg protein in 0.5 ml). The beads were washed with Tris buffer, containing 0.1% w/v βDDM, and then boiled at 90°C for 5 min to elute the complex. The proteins in samples were run on an SDS-PAGE gel and Western blotting was performed, with antibodies to the His-tag, to detect if the MtrD and MtrE prey proteins were present in the samples. Furthermore, the samples were subjected to MALDI-ToF PMF analyses, performed using a Voyager-DE™ STR BioSpectrometry™Workstation (Applied Biosystems, Warrington, UK). De-isotoped and calibrated spectra were then used to generate peak lists, which were searched using MASCOT (http://www.matrixscience.com) mass spectrometry database search software to identify the proteins. As a control, the GST protein was immobilized on GST column beads and tested for any interaction with the His-tagged prey proteins. No interaction of the His-tagged MtrD and MtrE prey proteins with GST was detected (data not shown).

### Cross-linking of MtrC and hairpin

NT-MtrC and MtrC hairpin proteins were first buffer exchanged with Na_2_HP0_4_, 100 mM NaCl, 10% v/v glycerol and 1 mM EDTA using mini chromatography column (Bio-RAD): 8 μM of each protein was incubated with 1 mM ethylene glycol bis-succinimidyl-succinate (EGS) at room temperature for 1 h. Then the cross-linking reaction was stopped with 100 mM Tris HCl, the sample was reduced with 50 mM DTT and mixed with 4× LDS sample buffer, boiled at 100°C for 10 min and separated on a 4–12% SDS-PAGE gradient gel with MOPS buffer.

### Homology modelling of MtrE and MtrD

The homology model for MtrE has previously been reported (Janganan *et al*., 2011a,b). Two independent homology models of MtrD were created using ESyPred3D (Lambert *et al*., 2002) and utilizing the MODELLER (Sali and Blundell, 1993) homology-modelling engine based on the high-resolution experimental structures of AcrB (2DHH.pdb) and MexB (2V50.pdb) respectively. Models were superposed and analysed using Coot (Emsley and Cowtan, 2004) and structural alignments executed with Espript (Gouet *et al*., 1999).
