# Supplementary material for: Tripartite efflux pumps: energy is required for dissociation, but not assembly or opening of the outer membrane channel of the pump
Source: Mol Microbiol. 2013 Apr 9;88(3):590–602. doi: 10.1111/mmi.12211 (PMC3664412; doi:10.1111/mmi.12211)
Supplement: Supplementary file 1 [file mmi0088-0590-SD1.pdf]

Supplementary Information and Data

TABLE S1:Strains and plasmids

Strains/ plasmids	***Description***	***Source***
***E. coli* strains**		
BL21(DE3)	*F⁻*, omp*T,* hsd*Sβ(rβ-mβ-),* dcm*,* gal*, (DE3) ton*	Stratagene
KAM3(DE3)	Antibiotic sensitive host strain used for expression of pDuet vectors – *ΔacrB*	Morita *et al*., 1998
(*ΔtolC*)TG1	Antibiotic sensitive host strain used for expression of pDuet vectors – *ΔtolC*	Nagakubo *et al*., 2002
(*ΔtolC*)TG1(DE3)	Antibiotic sensitive host strain used for expression of pDuet vectors – *ΔtolC*	Janganan *et al*., 2011a
(*ΔacrB*)C43(DE3)	Antibiotic sensitive host strain used for expression of pDuet vectors – *ΔacrB*	Gift from KM Pos.
**Plasmid cloning vectors**		
pET21a/pET24a	Expression of His-tagged proteins in *E. coli*	Novagen
pACYCDuet	Simultaneous expression of two proteins in *E. coli*	Novagen
**Plasmid constructs**		
pET21a-mtrE	*mtrE* cloned into pET21a(+) using NdeI-mtrE F and HindIII-mtrE R primers.	Janganan *et al*., 2011a
pET21a-mtrD	*mtrD* cloned into pET21a(+) using NdeI-mtrD F and HindIII-mtrD R primers.	Janganan *et al*., 2011a
pET21a-NT-mtrC	*mtrC* mutant, encoding a derivative truncated at position 34, cloned into pET21a(+) using NdeI-Δ34-mtrC F and XhoI-mtrC R primers.	Janganan *et al*., 2011a
pGEX6p-3 NT-MtrC (GST Tag)	*mtrC* mutant, encoding a derivative truncated at position 34, cloned into pGEX6P-3 using BamHI-Δ34-mtrC F and XhoI-mtrC R primers.	Janganan *et al*., 2011a
pET24a-gIII-NT-mtrC	*NT-mtrC* was cloned into pGEM-T using MtrC NT NcoI F and MtrC NT XhoI R primers; an *NcoI/XhoI* fragment was rescued and cloned into pBAD-gIIIC, from which *gIII-NT-mtrC* was amplified and ligated into pET24a(+).	This study
pET21a-mtrC hairpin	A DNA fragment encoding the α-helical hairpin domain of MtrCwascloned into pET21a(+) using	This study

	NdeI-mtrC hairpin F and NdeI-mtrC hairpin R primers.	
pET24a-gIII-mtrC hairpin	*mtrC-hairpin* was cloned into pGEM-T using MtrC NT NcoI F and MtrC NT XhoI R primers; an *NcoI/XhoI* fragment was rescued and cloned into pBAD-gIIIC, from which *gIII-hairpin* was amplified and ligated into pET24a(+).	This study
pACYCDuet-mtrE	*mtrE* cloned into MCSI of pACYCDuet using BamHI F and HindIII R primers.	This study
pACYCDuet-mtrD	*mtrD* cloned into MCS1 of pACYCDuet using BamHI F and SalI R primers.	This study
pACYCDuet-mtrC/mtrE	*mtrC* cloned into MCSI of pACYCDuet using BamHI-mtrC F and HindIII-mtrC R primers; *mtrE* cloned into MCS2 of pACYCDuet using NdeI-mtrE F and KpnI-mtrE R primers.	This study
pACYCDuet-mtrC hairpin/mtrE	*mtrE* cloned into MCS1 of pACYCDuet, using BamHI F and HindIII R primers, and MtrC hairpin cloned into MCS2, with a gIII-sequence-NdeI forward and XhoI reverse primers.	This study
pACYCDuet-mtrC hairpin/mtrD	*mtrD* cloned into MCSI of pACYCDuet, using BamHI F and SalI R primers, and MtrC hairpin cloned into MCS2, with gIII-sequence-NdeI forward and XhoI reverse primers	This study
pACYCDuet-mtrC/mtrD	*mtrC* cloned into MCSI of pACYCDuet, using BamHI-SalI F; and mtrD cloned into MCS2, using NdeI-XhoI primers.	Janganan *et al*., 2011a
pACYCDuet-mtrC/mtrD/mtrE	*mtrCmtrE* cloned into MCS1 of pACYCDuet using BamHI-mtrC F, EcoRI-mtrC-SD-R, EcoRI-mtrE -ATG F and SalI-mtrE R; *mtrD*cloned into MCS2 of pACYCDuet using NdeI-mtrD F and KpnI-mtrD R.	Janganan *et al*., 2011a

TABLE S2: primers for mtr*CDE* constructions

Constructs	Primers
pET21a-mtrE	MtrE NdeI F CATATGAATACTACATTGAAAACTACCTTGACCTCTGTTG<br>MtrE HindIII R AAGCTTTTTGCCGGTTTGGGTATCCCGTTTCAATCCGC
pET21a-mtrD	MtrD NdeI F CATATGGCTAAATTCTTTATCGACCGCCCCATTTTCG<br>MtrD HindIII R CTCGAGATATTGTTTATCGTCCGAACCGGTTATACCCG
pET21a NT-MtrC	MtrC NT NdeI F CATATGGGCTTTTTATGCTTCTAAGGCGATGCGTGCG<br>MtrC NT XhoI R CTCGAGTTTCGCTTCAGAAGCAGGTTTGGCTTCA
pGEX6p-3 NT-MtrC	MtrC NT BamHI F GGATCCGGCGGGCAGCCTGCGGGTCGGGAA<br>MtrC NT XhoI R CTCGAGTTATTTCGCTTCAGAAGCAGGTTTGGCTTCAGATGCCGTC
pET24agIII-MtrC NT	MtrC NT NcoI F CCATGGGGCTTTTTATGCTTCTAAGGCGATGCGTGCG<br>MtrC NT XhoI R CTCGAGTTTCGCTTCAGAAGCAGGTTTGGCTTCA<br>gIII NdeI F CATATGAAAAAACTGCTGTTCGCGATTCCG
pET21aMtrC Hairpin	MtrC hairpin F NdeICATATGATCGACAGTTCCACTTATGAAGC<br>MtrC hairpin R XhoI CTCGAGAATGCGCGAACGGTTCAGATTG
pET24a& pET21a - mtrC Hairpin	MtrC hairpin NcoI F CCATGGAGATCGACAGTTCCACTTATGAAGG<br>MtrC hairpin XhoI R CTCGAGAATGCGCGAACGGTTCAGATTG<br>gIII NdeI FCATATGAAAAAACTGCTGTTCGCGATTCCG
pACYCDuet-mtrE	MtrE BamHI F GGATCCGAATACTACATTGAAAACTACCTTG<br>MtrE HindIII R AAGCTTTTTGCCGGTTTGGGTATCCCGTTTCAATCCGC
pACYCDuet-mtrD	MtrD BamHI F GGATCCGGCTAAATTCTTTATCGACCGCCCCATTTTCG<br>MtrD SalI RGTCGACATATTGTTTATCGTCCGAACCGGTTATACCCG
pACYCDuet-mtrC/mtrE	MtrC BamHI F GGATCCGGCTTTTTATGCTTCTAAGGCGATGCGTGCG<br>MtrC SalI R GTCGACTTTCGCTTCAGAAGCAGGTTTGGCTTCAG<br>MtrE NdeI F CATATGAATACTACATTGAAAACTACCTTGACCTCTGTTG<br>MtrE KpnI R GGTACCTTTGCCGGTTTGGGTATCCCGTTTCAATCCGC
pACYCDuet-mtrC hairpin/mtrE	MtrE BamHI F GGATCCGAATACTACATTGAAAACTACCTTG<br>MtrE HindIII R AAGCTTTTTGCCGGTTTGGGTATCCCGTTTCAATCCGC<br>MtrC Hairpin gIIINdeI F CATATGAAAAAACTGCTGTTCGCGATTCCG<br>MtrC hairpin XhoI R CTCGAGAATGCGCGAACGGTTCAGATTG
pACYCDuet-mtrC/mtrD	MtrC BamHIF GGATCCGGCTTTTTATGCTTCTAAGGCGATGCGTGCG<br>MtrC SalI R GTCGACTTTCGCTTCAGAAGCAGGTTTGGCTTCAG<br>MtrD NdeI F CATATGGCTAAATTCTTTATCGACCGCCCCATTTTCG<br>MtrD XhoI R CTCGAGATATTGTTTATCGTCCGAACCGGTTATACCCG
pACYCDuet-mtrC/mtrD/mtrE	MtrC BamHI F GGATCCGGCTTTTTATGCTTCTAAGGCGATGCGTGCG<br>MtrC SD linker EcoRI R GAATTCTTATTATTCCTCTTATTTCGCTTCAGAAGCAGG<br>MtrE EcoRI F GAATTCATGAATACTACATTGAAAACT<br>MtrE HindIII R AAGCTTTTTGCCGGTTTGGGTATCCCGTTTCAATCCGC<br>MtrD NdeI F (MCS2) CATATGGCTAAATTCTTTATCGACCGCCCCATTTTCG<br>MtrD KpnI R GGTACCATATTGTTTATCGTCCGAACCG

Notes: sequences are 5' to 3'.

TABLE S3: Primers used for mutagenesis of *mtrE and mtrD*

MtrE Derivatives	Primers
E434K	For GCAGCTATTCGGCGAAAGGTGCGGCTTTG<br>Rev CAAAGCCGCACCTTTCGCCGAATAGCTGC
**MtrD Derivatives**	
D405K	For ATCGGCATCGTGGTCAAGGACGCGATTGTGGTG<br>Rev CACCACAATCGCGTCCTTGACCACGATGCCGAT
D406K	For CGGCATCGTGGTCGATAAGGCGATTGTGGTGG<br>Rev CCACCACAATCGCCTTATCGACCACGATGCCG
D405K/D406K	For ATCGGCATCGTGGTCAAGAAGGCGATTGTGGTGGTT<br>Rev AACCACCACAATCGCCTTCTTGACCACGATGCCGAT
K948E	For GTGATGGGTTTGAGTGCGGAGAACGCGATTCTGATTATC<br>Rev GATAATCAGAATCGCGTTCTCCGCACTCAAACCCATCAC

Notes: these primers were used to introduce mutations into the *mtrE* and *mtrD* genes in the expression vectors pET21a or pACYC; for the *mtrD* D405K/D406K/K948E triple mutant, the D405K/D406K mutations were first introduced and then the K948E mutation was introduced.

Fig. S1. The MtrCDE-pump is activated by the MtrC-hairpin.

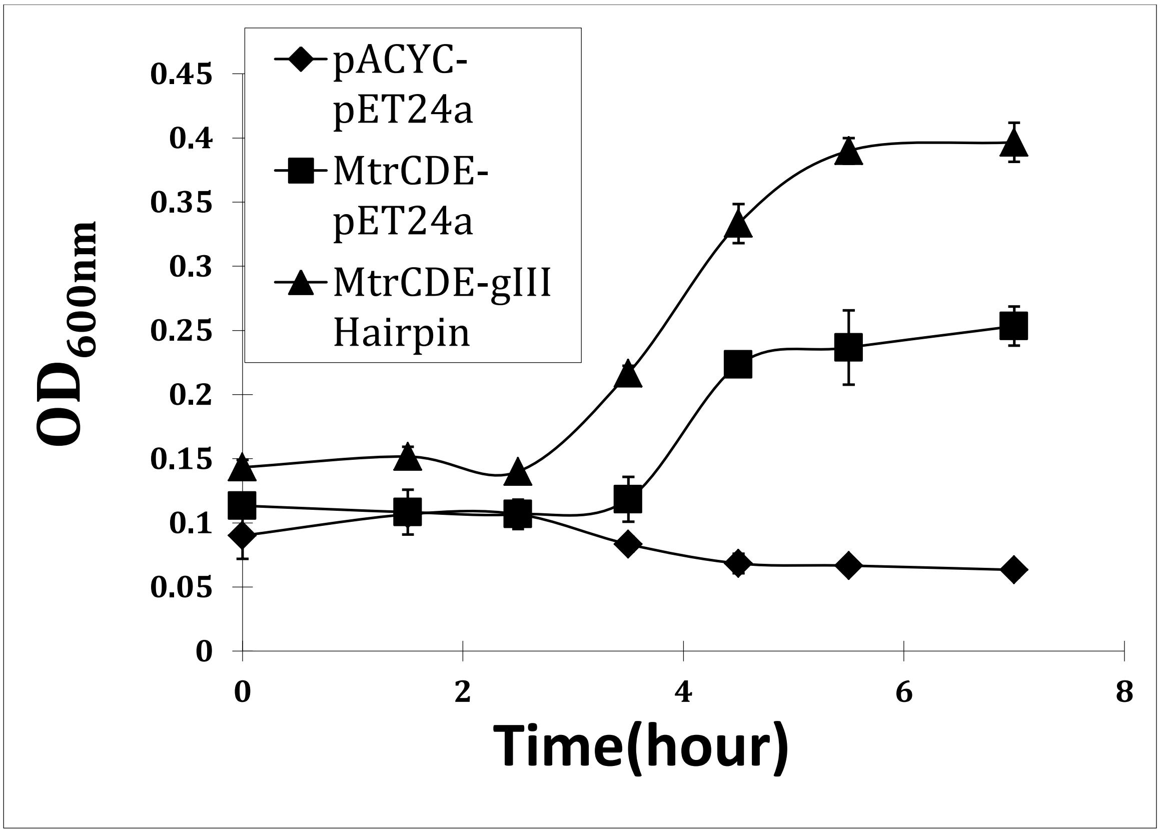

A series of curves showing the relative growth, measured as the OD_{600nm} over 7.5 hours, for *E. coli* cells KAM3(DE3)/pACYC/pET24a, Kam3(DE3)/pACYC-MtrCDE/pET24a and KAM3(DE3)/ pACYC-MtrCDE/pET24a-gIII-Hairpin with 64 μg/ml Nafcillin. Cells expressing MtrCDE with the gIII-Hairpin had an enhanced rate of growth compared to cells expressing only MtrCDE. Each bar is the average of at least 3 measurements, with error bars representing the standard deviation of these measurements from the average.

Fig. S2. Structural alignment of AcrB, MexB and MtrD.

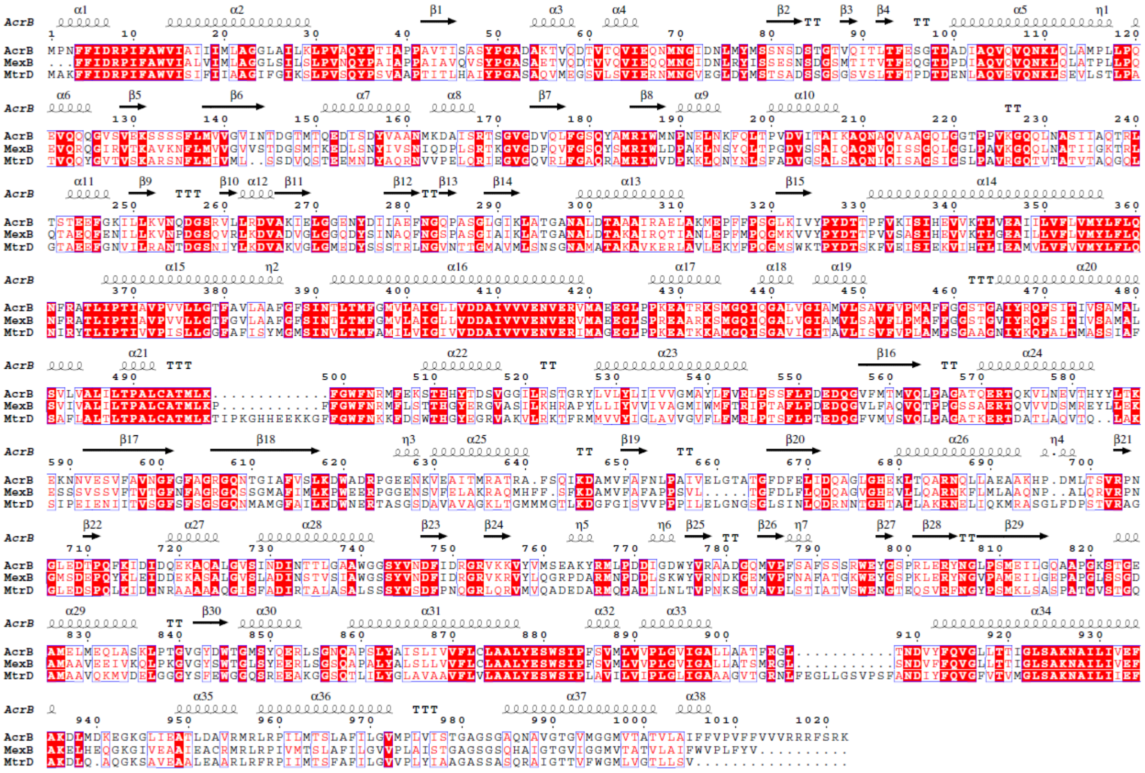

A structural alignment showing the secondary structural elements of AcrB (as per PDB 2DHH, chain A) was made with Espript.
